# Risk factor analysis of cognitive frailty in older adults with type 2 diabetes mellitus: a cross-sectional study

**DOI:** 10.3389/fnagi.2025.1667405

**Published:** 2025-09-25

**Authors:** Qingjuan Ren, Shu Niu, Lei Zhi, Hongfang Liu

**Affiliations:** ^1^Department of Geriatrics, Shijiazhuang People’s Hospital, Shijiazhuang, Hebei, China; ^2^Department of Endocrinology, Shijiazhuang People’s Hospital, Shijiazhuang, Hebei, China

**Keywords:** older adults, type 2 diabetes, cognitive frailty, cross-sectional study, frailty

## Abstract

**Background:**

Cognitive Frailty (CF), characterized by the co-occurrence of physical frailty and cognitive impairment without dementia, is a significant concern in older adults with Type 2 Diabetes Mellitus (T2DM). This study investigates the prevalence and risk factors of CF in this population, addressing the urgent need for early detection and intervention strategies.

**Methods:**

A cross-sectional study was conducted among 713 T2DM patients aged ≥65 years. Participants were assessed using the Clinical Dementia Rating (CDR) for cognitive function and Fried Frailty Phenotype (FP) criteria for physical frailty. CF was defined as CDR = 0.5 and FP score ≥ 3. Multivariable logistic regression identified independent risk factors, adjusting for demographic and clinical variables.

**Results:**

The prevalence of CF was 24.3%. Cerebrovascular disease (OR: 2.39, 95% CI: 1.44–3.97) and peripheral arterial disease (OR: 1.74, 95% CI: 1.03–2.95) were significant risk factors, while higher education (OR 0.85, 95% CI 0.76–0.94) and later diabetes onset (OR: 0.93, 95% CI: 0.88–0.98) were protective. Early-onset diabetes significantly increased CF risk regardless of education level.

**Conclusion:**

This study highlights a markedly increased risk of CF in older adults with T2DM, especially in individuals with CVD, PAD, low education levels, or early-onset diabetes. Notably, early diabetes onset may override the protective effects of cognitive reserve, emphasizing the critical need for early metabolic and vascular risk management in this high-risk population. These findings support integrated screening for cognitive and physical frailty in diabetes care to enable timely interventions.

## Introduction

Against the backdrop of global aging, the comorbidity of Type 2 Diabetes Mellitus (T2DM) and geriatric syndromes has emerged as a critical public health concern, with the synergistic deterioration of physical frailty and cognitive impairment posing particular challenges. Epidemiological evidence indicates that the prevalence of physical and cognitive decline in older adults with T2DM is 2–3 times higher than in non-diabetic populations (Hanlon et al., 2020; Srikanth et al., 2020; Xue et al., 2019). This dual frailty state not only exacerbates care dependency and healthcare expenditures but may also initiate a detrimental feedback loop, further compromising clinical prognosis (Kasajima et al., 2022).

Cognitive Frailty (CF), an emerging clinical syndrome in geriatrics, is defined by the co-occurrence of physical frailty and cognitive impairment in the absence of dementia. The majority of affected individuals ultimately develop both frailty and dementia. Substantial evidence demonstrates that CF markedly elevates the risk of detrimental health outcomes in elderly populations, including falls, functional impairment, increased hospitalization rates, and mortality (Hao et al., 2018; Nader et al., 2023).

Notably, CF is considered a dynamic and potentially reversible condition, highlighting the importance of early detection and intervention. Timely screening and targeted interventions may not only postpone dementia onset but also mitigate frailty-related functional decline. This cross-sectional study aims to identify independent risk factors for CF in elderly patients with T2DM, with the ultimate goal of improving early detection and reducing disability incidence.

## Materials and methods

### Research design

This hospital-based cross-sectional study consecutively enrolled T2DM patients aged ≥65 years from the endocrinology outpatient clinic between January 2025 and June 2025. We excluded patients with severe cardiovascular, mental, or musculoskeletal disorders. Following written informed consent and ethical approval (Shijiazhuang People’s Hospital IRB No. 2025137, in accordance with the Helsinki Declaration), all participants underwent a standardized assessment including: (1) demographic questionnaires and clinical data collection (2) Clinical Dementia Rating (CDR) for cognitive screening, and (3) Fried Frailty Phenotype (FP) criteria for physical frailty evaluation. CF was defined according to an international consensus as the co-existence of physical frailty (FP score ≥ 3) and mild cognitive impairment (CDR score = 0.5), in the absence of dementia (Cozza and Boccardi, 2025). Participants meeting these criteria were classified as having CF and proceeded to comprehensive risk factor analysis.

### Data collection methods

#### Basic demographic and clinical data collection

All participants completed a standardized questionnaire to collect demographic characteristics (age, sex, education level), diabetes history (age at diagnosis, duration). Comorbid conditions were systematically verified through medical record review, encompassing major cardiovascular diseases (coronary artery disease [CAD], cerebrovascular disease [CVD], and hypertension [HTN]) as well as diabetes-specific complications (diabetic nephropathy [DN], diabetic retinopathy [DR], diabetic peripheral neuropathy [DPN], and diabetic peripheral arterial disease [PAD]). This multidimensional data collection ensured thorough characterization of each participant’s clinical profile for subsequent analyses.

#### Cognitive function evaluation

Cognitive function was evaluated using the validated CDR scale, a standardized assessment tool that comprehensively examines six key functional domains: memory, orientation, judgment and problem-solving abilities, community activities participation, home and hobbies maintenance, and personal care capacity. Based on the evaluation, participants were assigned categorical scores ranging from 0 (indicating normal cognitive function) to 3 (representing severe dementia), with intermediate scores of 0.5 (questionable impairment), 1 (mild impairment), and 2 (moderate impairment) (Morris, 1993). Individuals with a CDR score of 0.5 (indicating mild cognitive impairment but not dementia) were enrolled in this study.

#### Physical frailty measurement

Frailty was evaluated using the FP criteria, which assesses five components: unintentional weight loss, slow walking speed, weak grip strength, low physical activity, and exhaustion. Each component is scored as 1 (“yes”) or 0 (“no”), with total scores ranging from 0 to 5 points. Based on the total score: 0 indicates non-frail, 1–2 indicates pre-frail, and 3–5 indicates frail status (Buta et al., 2016). For the present study, we specifically enrolled participants with FP scores ≥ 3, corresponding to the frail phenotype.

### Statistical analysis

Data were analyzed using SPSS 25.0 (IBM Corp, Armonk, NY). Continuous variables with normal distribution were presented as mean ± standard deviation, while categorical variables were reported as numbers (percentages). Comparisons between the CF group and the non-cognitive frailty group were performed using independent *t*-tests for continuous variables and chi-square or Fisher’s exact tests for categorical variables, as appropriate. Variables showing significant differences in these univariate analyses were subsequently included as potential influencing factors in a binary logistic regression model to identify independent risk factors for cognitive frailty. A two-tailed *P*-value < 0.05 was considered statistically significant.

## Results

**FIGURE 1:**
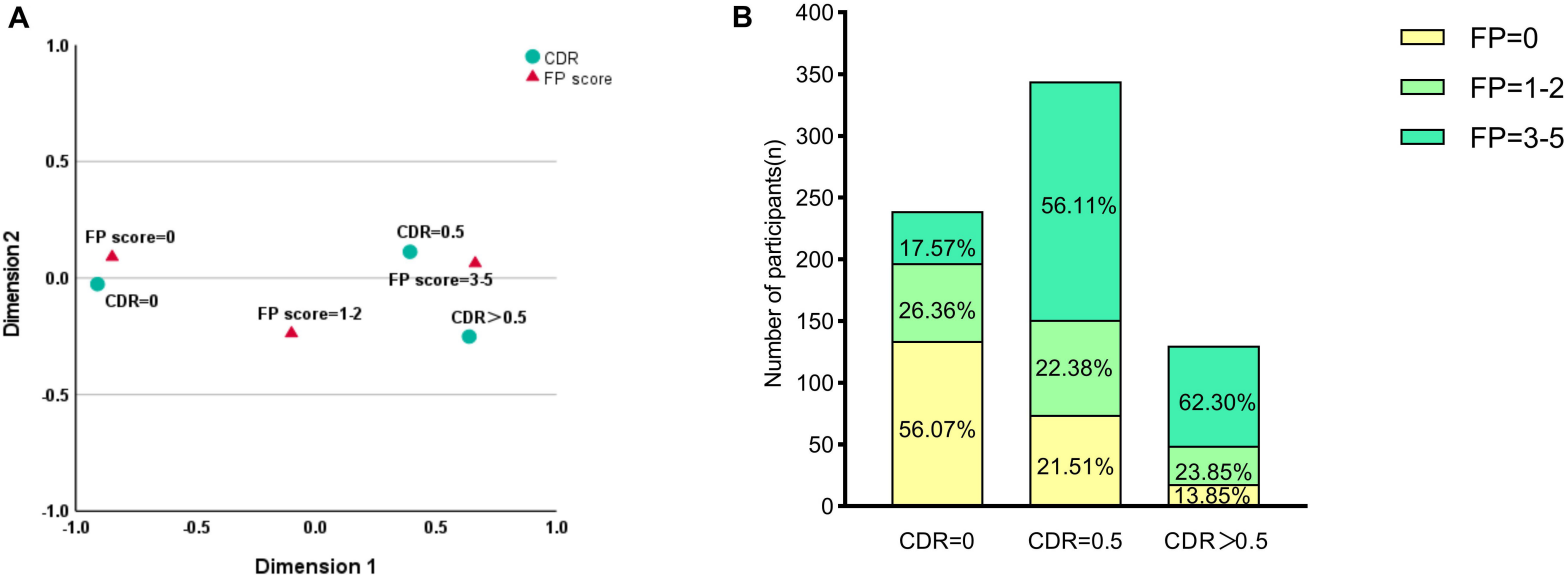
Correspondence analysis and distribution of Cognitive Dementia Rating (CDR) and Fried Phenotype (FP) scores. **(A)** Correspondence analysis plot illustrating the associations between cognitive status (CDR categories) and physical frailty (FP scores). The analysis revealed distinct clustering patterns: CDR = 0 correlated with FP = 0 (cognitively and physically normal), while CDR ≥ 0.5 clustered with FP = 3–5 (cognitively and physically impaired). **(B)** Stacked bar chart showing the prevalence of physical frailty phenotypes within each cognitive group. Values represent the percentage of participants within each CDR category. Most participants with CDR = 0 were physically robust (FP = 0; 56.07%), whereas the CDR = 0.5 group showed a high prevalence of pre-frailty (FP = 1–2; 56.11%). Among those with CDR > 0.5, 62.30% were classified as frail (FP = 3–5).

Among 713 older adults with T2DM, CF prevalence was 24.3% (173/713). Correspondence analysis revealed distinct CDR-FP score clustering: CDR = 0 with FP score = 0 (normal), and CDR ≥ 0.5 with FP score = 3–5 (impaired) (Figure 1A). Figure 1B presents the detailed distributions of CDR and FP scores in the study cohort. Compared to the 134 controls without frailty or cognitive impairment, CF patients demonstrated significantly lower education (*p* = 0.002), longer diabetes duration (*p* < 0.001), earlier onset (*p* < 0.001), and higher cardiovascular (*p* = 0.022) and peripheral arterial disease (*p* = 0.018) prevalence (Table 1).

**TABLE 1:** Baseline characteristics of participants.

Variables	Cognitive frailty (*n* = 173)	Non-cognitive frailty (*n* = 134)	*P*-value
Age (years)	72.98 ± 5.64	73.78 ± 5.41	0.214
Females, *n* (%)	101 (58.4)	76 (56.7)	0.816
Education (years)	8.54 ± 2.61	9.40 ± 2.23	0.002
T2DM duration (years)	15.51 ± 8.10	11.22 ± 5.65	0.000
Onset age of T2DM (years)	57.47 ± 8.78	62.56 ± 6.67	0.000
CVD, *n* (%)	94 (54.3)	55 (41)	0.022
CAD, *n* (%)	95 (54.9)	63 (47.4)	0.206
HTN, *n* (%)	76 (43.9)	52 (38.8)	0.414
DN, *n* (%)	81 (46.8)	63 (47)	1.000
DR, *n* (%)	83 (48)	66 (49.3)	0.908
DPN, *n* (%)	81 (46.8)	52 (38.8)	0.166
PAD, *n* (%)	75 (43.4)	40 (29.9)	0.018

T2DM, type 2 diabetes mellitus; CVD, cerebrovascular disease; CAD, coronary artery disease; HTN, hypertension; DN, diabetic nephropathy; DR, diabetic retinopathy; DPN, diabetic peripheral neuropathy; PAD, diabetic peripheral arterial disease.

**FIGURE 2:**
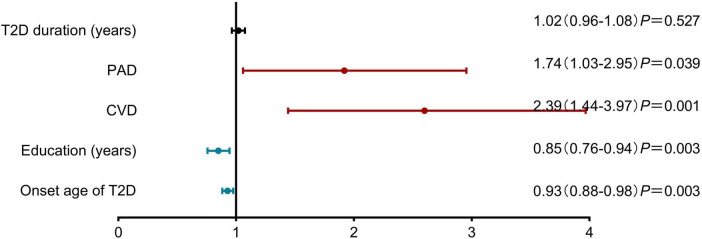
Risk factors associated with cognitive frailty in older adults with Type 2 Diabetes Mellitus. Results of Multivariable analysis are presented as adjusted odds ratios (OR) with 95% confidence intervals. Later diabetes onset (OR: 0.93, 95% CI: 0.88–0.98; *P* = 0.003) and higher education level (OR: 0.85, 95% CI: 0.76–0.94; *P* = 0.003) were identified as protective factors. Conversely, peripheral arterial disease (OR: 1.74, 95% CI: 1.03–2.95; *P* = 0.039) and cerebrovascular disease (CVD) (OR: 2.39, 95% CI: 1.44–3.97; *P* = 0.001) were significant risk factors. T2D duration was not significantly associated with cognitive frailty (OR: 1.02, 95% CI: 0.96–1.08; *P* = 0.527). CVD, cerebrovascular disease; PAD, diabetic peripheral arterial disease.

Multivariable analysis identified later diabetes onset (OR: 0.93, 95% CI: 0.88–0.98) and higher education (OR: 0.85, 95% CI: 0.76–0.94) as protective factors, whereas cerebrovascular disease (OR: 2.39, 95% CI: 1.44-3.97) and peripheral arterial disease (OR: 1.74, 95% CI: 1.03–2.95) independently associated cognitive frailty (Figure 2).

**FIGURE 3:**
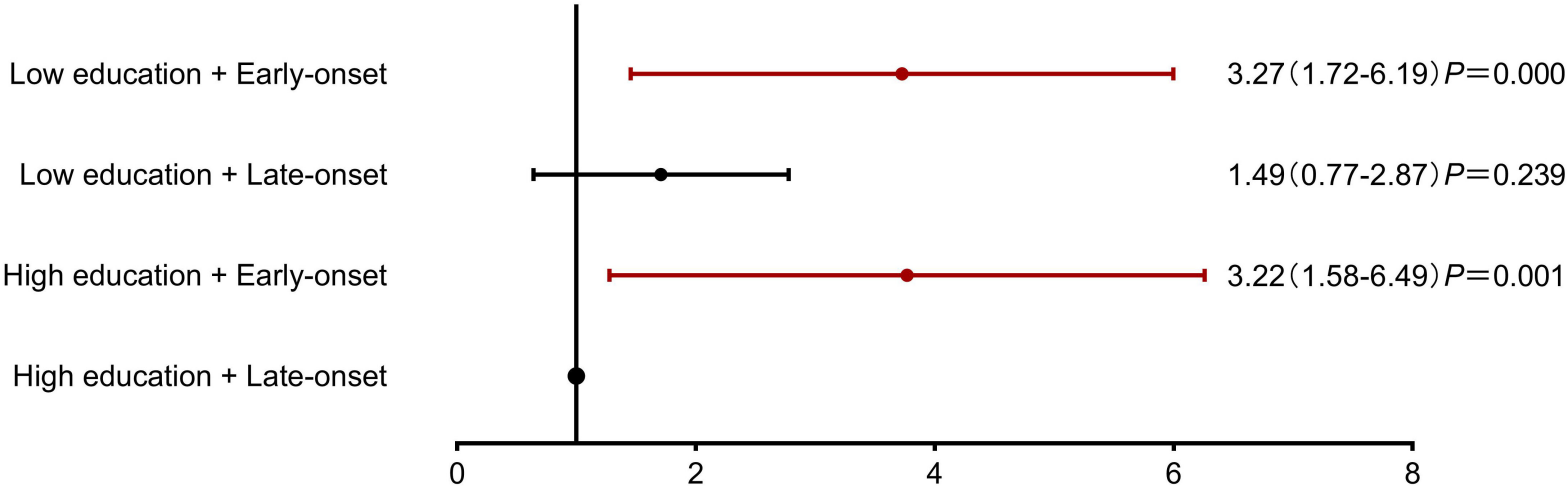
Association of educational attainment and diabetes onset age with Cognitive Frailty risk. Stratified analysis demonstrating the combined effects of education level and diabetes onset age on cognitive frailty risk. The reference group was high education (≥9 years) with late-onset diabetes (≥65 years). Both early-onset diabetes (<65 years) groups showed significantly increased risk, independent of education level (low education + early-onset: OR = 3.27, 95% CI: 1.72–6.19, *P* < 0.001; high education + early-onset: OR = 3.22, 95% CI: 1.58–6.49, *P* = 0.001). The low education + late-onset diabetes group showed a non-significant risk increase (OR = 1.49, 95% CI: 0.77–2.87, *P* = 0.239).

For stratified analysis, participants were categorized by: (1) education level (high: ≥9 years; low: <9 years) and (2) diabetes onset age (early: <65 years; late: ≥65 years), creating four distinct subgroups. After adjusting for cerebrovascular disease and peripheral arterial disease, both early-onset diabetes groups showed elevated cognitive frailty risk regardless of education level (high education + early-onset: OR: 3.22, CI: 1.58–6.49; low education + early-onset: OR: 3.27, CI: 1.72–6.19), while the low education + late-onset group demonstrated non-significant risk elevation (OR: 1.49, CI: 0.77–2.87) compared to the high education + late-onset reference group (Figure 3).

## Discussion

### Diabetes as an accelerator of cognitive-physical decline

This study reveals a notably high prevalence of CF (24.3%) among older adults with T2DM, which is substantially higher than the rate of 1.0%–4.4% reported in community-based populations (Nader et al., 2023). This finding underscores the role of diabetes as a significant accelerator of both physical and cognitive aging processes. Cognitive impairment and frailty represent two prevalent geriatric syndromes that frequently coexist. Frailty manifests as a complex physiological state marked by diminished physiological reserve and heightened vulnerability to stressors, arising from multisystem dysregulation (Kim and Rockwood, 2024). Cognitive impairment, often regarded as a prodromal stage of dementia, can severely compromise patients’ activities of daily living when left unaddressed. Emerging evidence suggests a bidirectional relationship between physical frailty and cognitive decline, supported by shared pathophysiological mechanisms including chronic low-grade inflammation, oxidative stress, mitochondrial dysfunction, and endocrine dysregulation (Kim and Rockwood, 2024; Pan and Ma, 2024). In T2DM patients, these processes are exacerbated by insulin resistance, suboptimal glycemic control, and microvascular damage, which collectively promote metabolic disturbances and substantially elevate the risk of concurrent cognitive impairment and frailty (Zhang et al., 2023). Notably, neuroimaging studies have identified early neurodegenerative changes in frail individuals, characterized by cerebral atrophy and amyloid-β (Aβ) deposition before overt cognitive symptoms (Cipolli et al., 2024). CF represents a critical window for clinical intervention when the condition may still be reversible. Targeted exercise and nutritional interventions have shown efficacy in mitigating CF progression during this pivotal stage (Reuben et al., 2024; Yoon et al., 2018; Zhang et al., 2023). This highlights the importance of early screening and preventive strategies for this vulnerable population.

### Vascular factors may be risk factors for CF

Logistic regression analysis identified CVD and PAD as independent risk factors for CF in T2DM patients. This finding underscores the pivotal role of vascular pathology in mediating cognitive decline in diabetes. Accumulating evidence positions CVD as a major contributor to dementia, with vascular cognitive impairment (VCI) accounting for 20%–40% of all dementia cases and frequently coexisting with neurodegenerative processes. The multifactorial etiology of VCI encompasses small vessel disease (SVD), large artery atherosclerosis, cerebral hemorrhage, cardioembolic events, and other stroke subtypes. These vascular insults impair cognition through mechanisms including cerebral hypoperfusion, blood-brain barrier disruption, endothelial dysfunction, neuroinflammation, and immunosenescence (Rundek et al., 2022; Zlokovic et al., 2020). Notably, cerebral SVD represents the most prevalent substrate of vascular dementia (contributing to approximately 20% of dementia cases), with aging and T2DM being its prominent risk factors (Markus and Joutel, 2025). Neuroimaging in diabetic patients consistently demonstrates characteristic SVD markers, including lacunar infarcts, white matter hyperintensities, cerebral microbleeds, and cortical microinfarcts (Cui et al., 2024). Importantly, vascular dysfunction also drives physical frailty progression in diabetes through macrovascular complications, microcirculatory impairment, and endothelial damage that collectively compromise tissue perfusion (Sinclair and Abdelhafiz, 2023).

These pathophysiological insights warrant comprehensive vascular assessment in T2DM patients, coupled with cognitive and frailty screening. Aggressive vascular risk factor modification - including optimized blood pressure control, statin therapy, and antiplatelet regimens when indicated - should be integrated into standard diabetes care to potentially mitigate CF risk, though further prospective studies are needed to validate these therapeutic approaches.

### Protective role of education and later diabetes onset

The logistic regression analysis demonstrated that higher educational attainment was significantly associated with reduced risk of CF, supporting the cognitive reserve hypothesis. This protective effect may stem from enhanced neural efficiency developed through educational experiences during critical neurodevelopmental periods in childhood and adolescence (Reuben et al., 2024; Stern et al., 2020). These findings suggest that cognitive training and lifelong learning interventions could potentially mitigate frailty progression in diabetic populations.

Additionally, later onset of T2DM emerged as an independent protective factor against CF. This association appears to be mediated by the substantially higher incidence of both macrovascular and microvascular complications in early-onset compared to late-onset T2DM patients (Magliano et al., 2020). Such complications serve as key drivers of cognitive impairment and frailty, explaining why early-onset T2DM shows stronger associations with all-cause dementia, Alzheimer’s disease dementia, and stroke risk (Wang and Liu, 2021). These results highlight education as a modifiable protective factor while identifying early diabetes onset as an important risk marker in CF development.

Subgroup logistic regression analyses revealed that the early-onset diabetes group exhibited significantly higher CF risk compared to the high education + late-onset group, regardless of educational attainment. However, no increased risk was observed in the low education + late-onset group, suggesting that the detrimental effects of early-onset diabetes may override the protective benefits conferred by higher education. These findings highlight the particularly strong association between early-onset diabetes and CF development. Furthermore, the results emphasize that preventing early-onset diabetes may serve as a crucial intervention point for reducing the risk of CF in older adults.

This study provides important evidence for the clinical management of diabetes-associated CF. For high-risk patients with early-onset diabetes, low educational attainment, or comorbid cerebrovascular/peripheral vascular diseases, regular monitoring of cognitive and physical function combined with active metabolic control and enhanced vascular protection may help mitigate CF risk. However, further research is needed to determine whether exercise training and nutritional support can effectively improve CF outcomes.

Given that CF reflects a pathological process accumulated over many years, the present study did not incorporate data on short-term clinical indicators such as glycemic control (HbA1c) or medication use, in alignment with the temporal nature of the exposures under investigation.

### Limitations and future directions

While this study offers valuable insights, several limitations should be considered. First, the cross-sectional design prevents causal inferences, and although early-onset T2DM demonstrated a strong association with CF, the temporal and causal nature of this relationship cannot be established from our data. Second, the use of CDR = 0.5 to define CF, though standard, may not fully exclude prodromal dementia due to inherent scale limitations, meaning our findings characterize a high-risk group consistent with the CF construct. Third, as a single-center study recruiting from an endocrinology clinic, our sample may overrepresent severe T2DM cases, limiting generalizability to community or primary care populations. Future studies should employ longitudinal designs, incorporate biomarkers, and validate findings in multi-center community cohorts.

## Conclusion

This study highlights a markedly increased risk of CF in older adults with T2DM, especially in individuals with CVD, PAD, low education levels, or early-onset diabetes. Notably, early diabetes onset may override the protective effects of cognitive reserve, emphasizing the critical need for early metabolic and vascular risk management in this high-risk population. These findings support integrated screening for cognitive and physical frailty in diabetes care to enable timely interventions.

## Data Availability

The original contributions presented in this study are included in this article/supplementary material, further inquiries can be directed to the corresponding author.
